# CT-imaging features of renal epithelioid angiomyolipoma

**DOI:** 10.1186/s12957-015-0700-9

**Published:** 2015-09-22

**Authors:** Ying Liu, Fangyuan Qu, Runfen Cheng, Zhaoxiang Ye

**Affiliations:** Department of Radiology, Tianjin Medical University Cancer Institute and Hospital, National Clinical Research Center of Cancer, Key Laboratory of Cancer Prevention and Therapy, 300060 Tianjin, China; Department of Pathology, Tianjin Medical University Cancer Institute and Hospital, National Clinical Research Center of Cancer, Key Laboratory of Cancer Prevention and Therapy, Tianjin, China

**Keywords:** Angiomyolipomas, Renal mass, Epithelioid angiomyolipoma, Computed tomography

## Abstract

**Background:**

The aim of this study was to describe the computed tomography (CT)-imaging features of renal epithelioid angiomyolipomas (E-AMLs) to understand and recognize this new category of renal tumors.

**Methods:**

Institutional review board approval was obtained for this retrospective study. Clinical data and preoperative CT images of 11 cases of E-AML were retrospectively analyzed. All patients had unenhanced and tri-phase dynamic enhanced CT examination. CT-imaging features including tumor size, existence of fat and calcification, enhancement degree, enhancement pattern, and enhancement heterogeneity were evaluated.

**Results:**

The patients were ten women and one man. The size of tumor ranged from 1.8 to 10.3 cm. All of them had distinct edges; one had a lobulated appearance, ten had bulging contour of the involved kidney, and four lesions had intratumoral fat. Eight of the E-AMLs demonstrated hyper-attenuation, two as iso-attenuation, and one as hypo-attenuation compared with renal parenchyma on unenhanced CT images. Contrast-enhanced CT features were markedly heterogeneous in eight cases (73 %). The predominant enhancement pattern was rapid wash-in to slow wash-out (91 %).

**Conclusions:**

The radiological appearance of most E-AMLs has a tendency to be hyper-attenuated on precontrast CT with or without fat component and demonstrates a rapid wash-in to slow wash-out dynamic enhancement pattern.

## Background

Angiomyolipoma (AML) is a histologically complex mesenchymal tumor composed of varying proportions of dysmorphic blood vessels, adipose tissue, and spindled smooth muscle cells [1]. It is a member of the perivascular epithelioid cell tumor (PEComa) family, which additionally includes lymphangiomyomatosis, clear cell “sugar” tumor of the lung, and morphologically and immunohistochemically related tumors at other sites [2]. AML comprises 2.0 to 6.4 % of all renal tumors and is most commonly benign in behavior [3]. Renal epithelioid angiomyolipoma (E-AML) is a rare variant of AML, which is composed of a prominent epithelioid component, with spindle and giant cells, and contains none or a minimal amount of adipose tissue [4]. Unlike classic AML, which follows a benign course, this rare subtype may exhibit aggressive biology, including local recurrence and metastasis [5, 6]. Therefore, the current World Health Organization Classification of Renal Neoplasms regards E-AML as “a potentially malignant mesenchymal neoplasm.” Although the imaging features of classical AML are well described in the radiology literature, the imaging appearance of E-AML has been much less well reported because of the rarity of this entity [5–10]. Herein, we retrospectively reviewed the imaging findings of E-AML as obtained using computed tomography (CT).

## Methods

### Patients

Institutional review board approval was obtained at our institution (Tianjin Medical University Cancer Institute and Hospital, Tianjin, China) for the present retrospective study. The requirement for informed consent was waived. We performed a search of our hospital’s pathology computer database for cases that occurred between May 2008 and December 2013 by using the search terms “nephrectomy,” “renal epithelioid angiomyolipomas,” and “angiomyolipoma, epithelioid.” We identified 15 consecutive patients who had undergone nephrectomy of renal epithelioid angiomyolipomas. The inclusion criteria were patients who underwent abdominal CT with unenhanced and tri-phase dynamic contrast enhanced within 1 month before surgery. Three patients who had abdominal CT scans from another hospital and one patient who had undergone only enhanced CT were excluded. Finally, 11 patients were enrolled in this study. The pathological proof of E-AML was obtained by surgical resection for all cases, and for the present study, E-AML was defined as tumors that were composed of predominantly epithelioid cells with abundant cytoplasm, vesicular nuclei, and prominent nucleoli. Immunohistochemical staining for the antibodies of HMB45, SMA, vimentin, CD34, and cytokeratin was also used to confirm the diagnosis in three cases.

### CT-scanning protocol

Abdominal CT examinations were performed by using one of two MDCT systems (Somatom Sensations 64, Siemens, Erlangon, Germany; High Speed Advantage, LightSpeed 16, GE, Waukesha, WI, USA). For the 16-detector scanner, scanning parameters were as follows: 120 kVp; 300–350 mA, pitch of 0.939; and slice thickness of 1.25 mm. For the 64-detector scanner, scanning parameters were as follows: 120 kVp with tube current adjusted automatically, pitch of 0.939, and slice thickness of 1.5 mm. Images were obtained from the top of the kidneys to pubis. Unenhanced CT scan was performed before administration of intravenous contrast material injection, and then, 80–100 ml non-ionic iodine contrast material (Iohexol, Omnipaque, GE Company, Shanghai, China) at a concentration of 300 mg iodine/ml was injected into the antecubital vein at a rate of 2.5 ml/s using a mechanical injector. Arterial, venous, and delayed-phase CT was obtained after initiation of intravenous contrast medium injection, acquired with delay of 28–30, 60–70, and 120 s, respectively.

### Image analysis

All CT images were reviewed by two radiologists, who were not informed beforehand of the pathologic findings. Decisions on tumor characteristics were reached by consensus. Tumor size, location, existence of fat and calcification, and enhancement heterogeneity were evaluated. For the detection of fat attenuation, a pixel analysis was also used for unenhanced CT images. Homogeneity was defined as being present when more than 90 % of the area was occupied by the same attenuation value, as ascertained by visual inspection. Precontrast attenuation was classified as hypo-attenuation, iso-attenuation, and hyper-attenuation by comparison with the attenuation of surrounding renal parenchyma by visual assessment. CT attenuations of unenhanced, arterial, venous, and delayed-phase were measured in all cases. The central slice which could manifest the largest part of tumor was selected then a region of interest (ROI) was drawn as large as possible to cover the most solid portion of the tumor, avoiding fat component, necrotic, or haemorrhagic areas. This procedure was repeated three times, and mean CT attenuation was obtained by average. The degree of enhancement of each phase was measured by calculating the difference in mean attenuation value between the arterial phase and unenhanced scan, venous phase and unenhanced scan, and delayed phase and unenhanced scan.

## Results

The mean age at presentation of these 11 patients was 48.2 years with a female-to-male ratio of 10:1. None of the patients are associated with tuberous sclerosis. Presenting symptoms and signs include flank pain and palpable abdominal mass in four patients, while others are all asymptomatic and found incidentally.

**Fig. 1:**
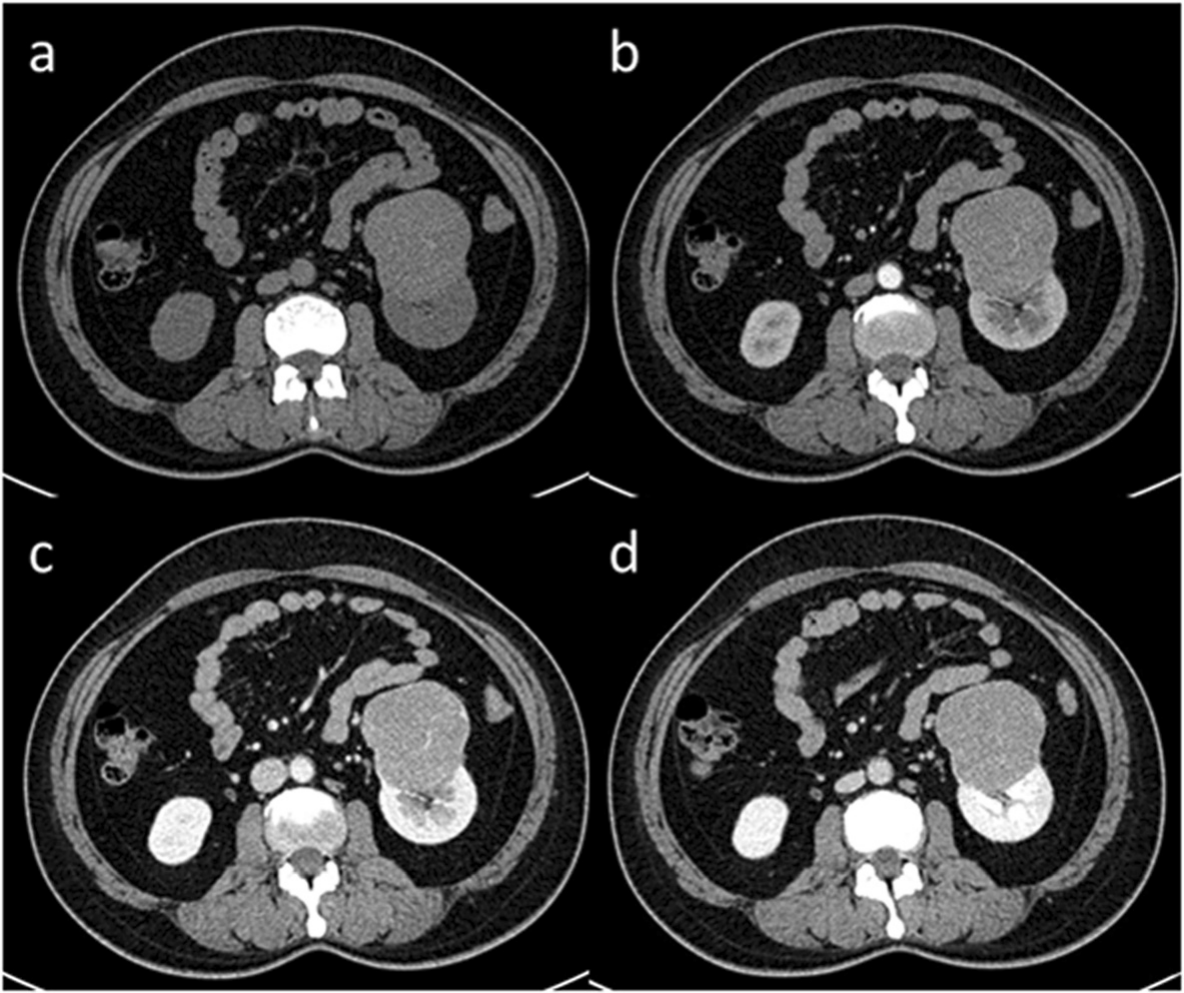
A 45-year-old woman with E-AML. The patient had no clinical symptoms. A renal mass was found during physical examination. Non-contrast axial CT image (**a**) demonstrates a hyper-density lesion with lobulated appearance in the lower renal pole of the left kidney. Contrast-enhanced CT images of arterial (**b**), venous (**c**), and delayed-phase (**d**) demonstrate relatively homogeneous enhancement

**Fig. 2:**
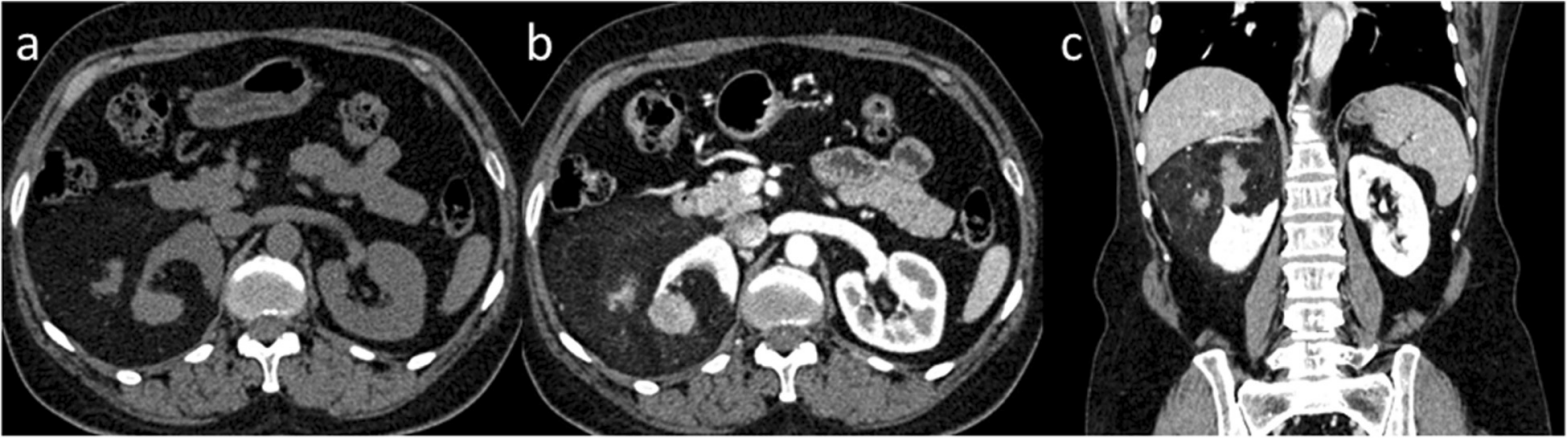
Macroscopic fat containing E-AML in a 62-year-old woman. Non-contrast axial CT image (**a**) demonstrates a slight hyper-density mass in the right kidney with abundance of fat and large amounts of soft tissue. Contrast-enhanced CT image of venous phase (**b**) and coronal reconstruction image of delayed-phase (**c**) demonstrate heterogeneous enhancement

**Fig. 3:**
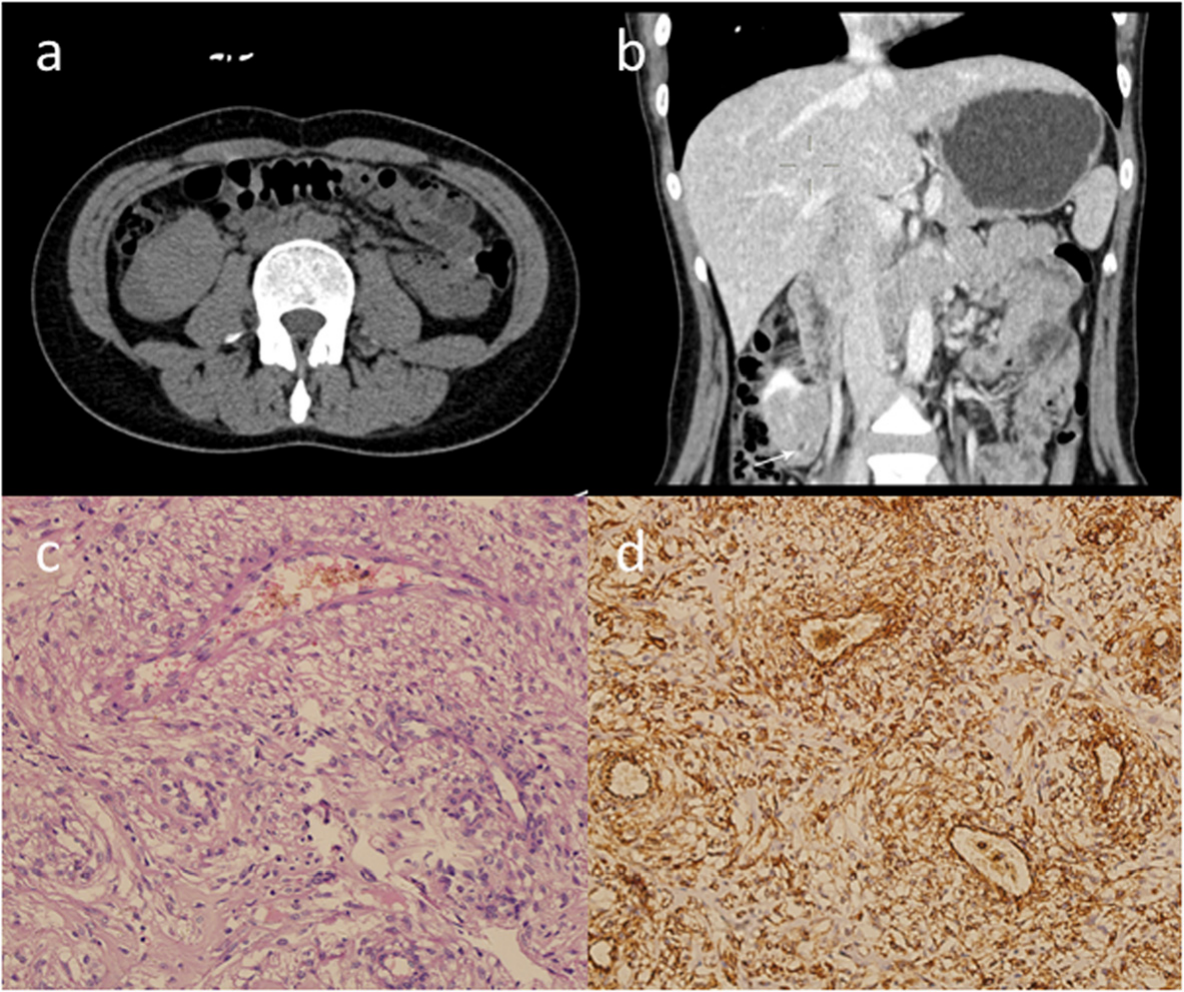
Minimum fat containing E-AML in a 35-year-old woman. Non-contrast axial CT image (**a**) demonstrates a hyper-density mass with distinct edge. Coronal reconstruction image of delayed-phase (**b**) demonstrates heterogeneous enhancement, and small foci of fat could be identified (*arrow*). Microscopically (**c**), the tumor is composed of thick-walled blood vessels, adipose tissue, and epithelioid cells (HE staining, original magnification ×200). Tumor cells show strong immunoreaction with antibody to HMB-45 (**d**) (immunohistochemical staining, original magnification ×200)

**Fig. 4:**
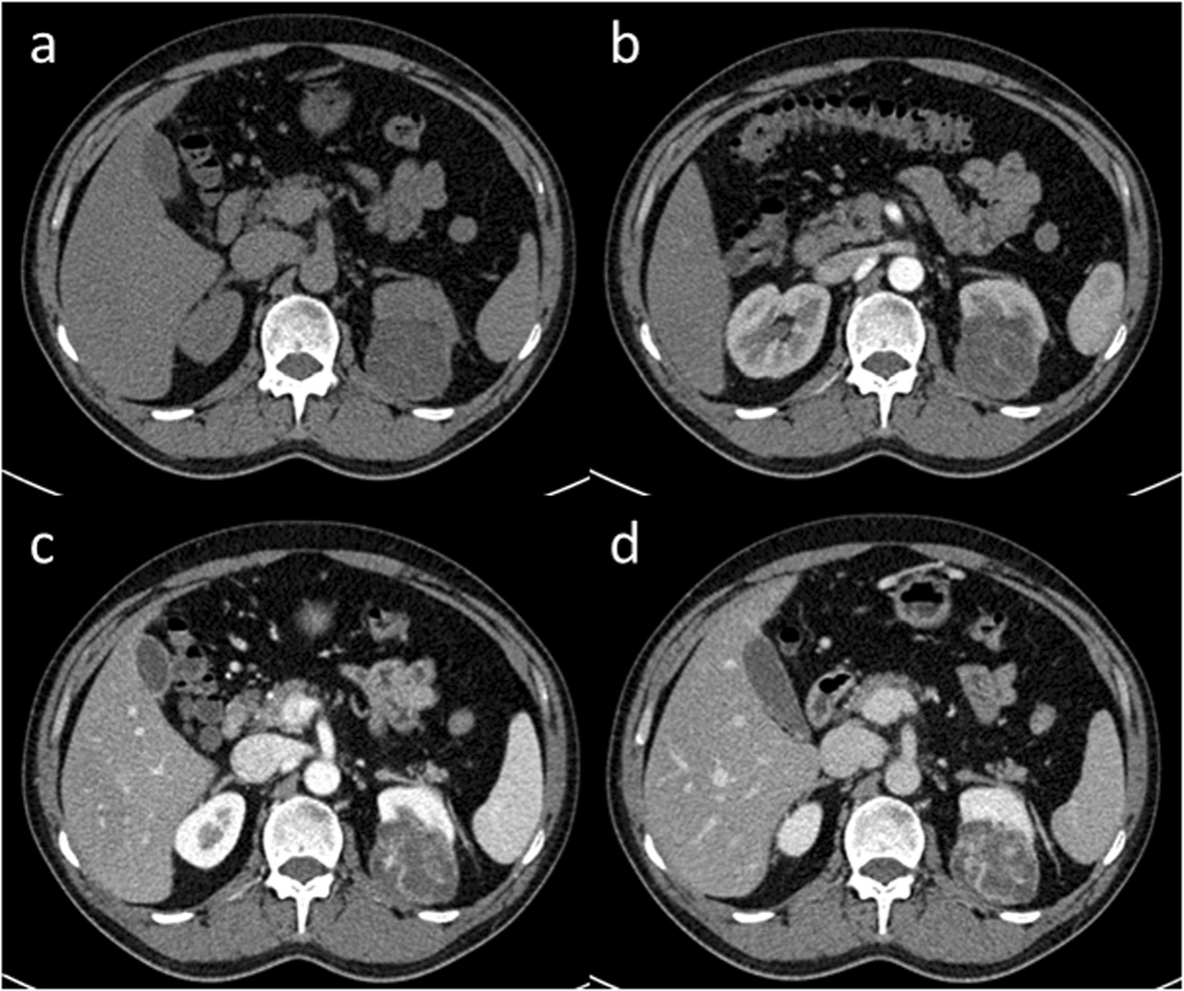
A 41-year-old male with an incidentally discovered left-sided renal mass. Axial non-contrast image (**a**) demonstrates an exophytic mass with hypo-density arising from the posterior aspect of the left kidney. Contrast-enhanced CT images of arterial (**b**), venous (**c**), and delayed-phase (**d**) demonstrate progressive heterogeneous enhancement with enlarged blood vessels presented in the lesion

Clinical and CT features of all patients were listed in Table 1. The renal lesions were in the right kidney in seven cases and the left in four cases. The lesions were located in the upper renal pole in five cases and lower renal pole in six cases. The median tumor diameter was 4.4 cm, ranging from a minimum diameter of 1.8 cm to maximum diameter of 10.3 cm. All of them had distinct edges; ten of them exhibited exophytic growth, and the maximum diameter of the tumor was beyond the expected kidney contour; one case had a lobulated appearance (Fig. 1). Intratumoral fat was identifiable with CT in four cases, among them one with a large quantity of fat (Fig. 2) and three with small foci of fat (Fig. 3). None of them had calcification. Eight cases were hyper-attenuation, two were iso-attenuation, and one was hypo-attenuation (Fig. 4) by comparison with the renal parenchyma on unenhanced CT images. The mass showed relatively homogeneous enhancement in three cases, while heterogeneous enhancement in eight cases (Fig. 4). The CT attenuation increased to different extensions after the injection of contrast medium, and at the venous phase, the degree of enhancement was greater than 20 HU for all of them (100 %) and exceeded 60 HU in eight cases (73 %) compared with unenhanced CT images. At the delayed phase, the CT attenuation decreased for ten cases (91 %), while only one case still had a little increase (Fig. 4). Evaluation of the pattern of dynamic enhancement revealed that ten lesions were categorized as “rapid wash-in and slow wash-out” (wash-in enhancement pattern means CT attenuation increases from unenhanced to arterial and venous phase; wash-out enhancement pattern means CT attenuation decreases from venous to delayed phase) and the other one as progressive enhancement which was characterized as CT attenuation increased steadily from the arterial to delayed phase. The border between the tumor and normal kidney was distinct in all cases. Additional classical AML could be found in one case. There was no imaging evidence of lymph node enlargement and vascular invasion in any of our cases.

**Table 1:** Clinical and CT features of 11 patients with renal epithelioid angiomyolipoma (E-AML)

Case	Age/sex	Location	Size (cm)	Fat	Calcification	Enhancement heterogeneity	CT attenuation (HU)			
							Unenhanced	Arterial phase	Venous phase	Delayed phase
1	51/F	Right/lower	2.1	No	No	Heterogeneous	41 ± 5.4	94 ± 6.9	106 ± 8.2	80 ± 5.7
2	55/F	Left/lower	1.8	No	No	Heterogeneous	44 ± 6.5	86 ± 5.5	155 ± 9.4	129 ± 8.6
3	45/F	Left/lower	6.1	No	No	Homogeneous	47 ± 6.1	69 ± 7.3	81 ± 7.9	75 ± 6.5
4	23/F	Right/upper	2.8	Yes	No	Heterogeneous	47 ± 5.8	103 ± 8.4	133 ± 9.6	109 ± 8.7
5	41/M	Left/upper	4.4	No	No	Heterogeneous	17 ± 1.7	29 ± 2.3	44 ± 3.5	54 ± 3.2
6	50/F	Left/upper	2.1	No	No	Homogeneous	46 ± 4.7	88 ± 6.3	124 ± 8.5	95 ± 8.2
7	55/F	Right/lower	3.3	No	No	Homogeneous	49 ± 5.9	96 ± 6.8	126 ± 6.1	102 ± 7.4
8	62/F	Right/upper	10.3	Yes	No	Heterogeneous	44 ± 5.6	82 ± 7.3	116 ± 8.9	101 ± 7.8
9	35/F	Right/lower	4.9	Yes	No	Heterogeneous	45 ± 4.8	117 ± 8.2	121 ± 9.5	115 ± 8.5
10	65/F	Right/upper	6.8	Yes	No	Heterogeneous	38 ± 4.1	77 ± 6.0	99 ± 5.4	87 ± 5.2
11	48/F	Right/lower	3.8	No	No	Heterogeneous	43 ± 4.8	69 ± 5.3	83 ± 6.2	76 ± 6.0

The pathological evaluation revealed epithelioid, short spindle cells with eosinophilic cytoplasm, large and deeply stained nuclei, and dense arrangement of tumor cells with patchy necrosis (Fig. 3). Immunohistochemical studies in three cases demonstrated that the tumor cells were positive for CD34, vimentin, SMA, and HMB-45, but negative for S-100 (Fig. 3).

One patient was followed up after surgery for 29 months and no obvious sign of recurrence or metastasis was found on follow-up CT images. Ten patients did not receive imaging follow-up because the diagnosis at the time was thought to be benign.

## Discussion

E-AML of the kidney occurs predominantly in females [3, 7, 9, 10], and according to Aydin et al. [3], patients with E-AML are younger than classical AML patients (38.6 vs. 52.3 years). In this study, the mean age at presentation was 48.2 (range, 23–65) years, and female-to-male ratio was 10:1. The differences in the reported mean age and gender ratio are likely due to the small number of cases reviewed in these series.

Like other AMLs, E-AMLs can occur in patients both with and without tuberous sclerosis. One of eight E-AMLs (12.5 %) described by Tsukada et al. [7] and two of nine cases (22.2 %) published by Froemming et al. [9] were seen in patients with tuberous sclerosis complex (TSC). Tsai et al. [11] found that approximately half of E-AML cases are associated with the TSC, all contained minimal to no fat, and all demonstrated a positive reaction to premelanosome antigens (PMAs). Cysts and multiple AMLs are the most common renal manifestation in TSC [12, 13]. In our series, only one case had additional classical AML; however, no evidence of tuberous sclerosis syndrome was documented.

In contradistinction to typical AMLs which have relatively characteristic imaging findings, E-AMLs often mimic renal cell carcinoma (RCC), renal sarcoma, or AML with minimal or absent fat on imaging evaluation, which may lead to incorrect diagnosis. To date, the imaging appearance of E-AML has been much less well reported than the histopathologic features since most of the imaging findings are individual reports. Tsai et al. [11] reported that none of the five lesions contained fat components (<−20 HU) or calcification. He also performed an analysis of the literature since 1998 and found that no fat component was described in any of the other 15 cases of E-AML with reported imaging findings. In another study conducted by Tsukada et al. [7], no fat component was detected on unenhanced CT images, T1-weighted images, T2-weighted images, or chemical fat suppression images in any of the eight E-AML cases. However, in other studies, fat was seen in the tumor at imaging [9, 14]. In this study, four lesions had components of fat identifiable at thin CT images, accounting for 36 % of all cases, and one of them had a large quantity of fat thus misdiagnosed as AML at preoperative imaging diagnosis. Most of the literatures that focused on imaging findings demonstrated that the characteristic imaging features of E-AML include higher density (than normal renal parenchyma) at unenhanced CT, bulging contour of the affected kidney, markedly heterogeneous enhancement, large lesion size on presentation, and complete capsule with distinct edges, occasionally with regional lymph node metastases [9–11]. All of the cases in our series had distinct edges; ten of them exhibited exophytic growth (91 %) and eight of them showed hyper-attenuation (73 %) on unenhanced CT images. The higher density at unenhanced CT has been ascribed to the densely packed epithelioid muscle component [15, 16]. The amount of enhancement described varied among different studies [7, 9, 10]. A recent study reported E-AMLs can present as heterogeneously or homogeneously enhancing solid masses or as multilocular cystic masses [7]. In the present study, most of the lesions showed heterogeneous enhancement (73 %), among which the presence of enlarged blood vessels could be seen in two cases, while the others showed relatively homogeneous enhancement (27 %). The predominant enhancement pattern is categorized as “rapid wash-in and slow wash-out,” which is in accordance with a previous study [10]. This phenomenon is thought to correlate with pathological descriptions: abundance of abnormal vessels, higher cellular density and decreased tumor stroma, presence of complete capsule, and lack of draining vessels.

The final diagnosis of E-AML will depend on the histopathological examination, especially immunohistochemistry. E-AML stains strongly for melanoma-associated markers, particularly HMB-45 (human melanoma, black) and negative for epithelial markers and S-100. Compared to E-AML, RCCs are positive for epithelial markers and negative for melanocytic markers and S-100 [2].

The current World Health Organization Classification of Renal Neoplasms regards E-AML as “a potentially malignant mesenchymal neoplasm” with adverse outcomes in approximately one third of cases. In a recent meta-analytical study in which 69 well-documented cases of epithelioid AML in the literature were reviewed, the malignancy rate was found to be 38 % [17]. However, given the relatively low number of cases thus far in the literature and variability in pathologic criteria for designating a tumor E-AML, the natural course remains uncertain but suggests that a significant minority of these tumors act in a malignant manner. In the present case, one patient was followed up after surgery for 29 months and no obvious sign of recurrence or metastasis was found on follow-up CT images.

The present study had several limitations. First, the number of cases was small. Thus, further evaluations using a large number of cases from multicenters are necessary to confirm our findings. Second, there was a limited spectrum of images available for each patient, and fat-sensitive imaging techniques, such as chemical shift suppression images, were not obtained. Finally, most patients did not receive imaging follow-up because the diagnosis at the time was thought to be benign.

## Conclusions

Most E-AMLs tend to present as a solid lesion with hyper-density on unenhanced CT with or without fat component and demonstrate rapid wash-in to slow wash-out dynamic enhancement pattern. Whenever a fatty renal mass contains large amounts of soft tissue, the possibility of E-AML should be included in the differential diagnosis.

### Consent to publish

Written informed consent was obtained from the patients for publication of this paper and any accompanying images.

## Abbreviations

AML: angiomyolipoma
CT: computed tomography
E-AML: epithelioid angiomyolipoma
PMAs: premelanosome antigens
RCC: renal cell carcinoma
ROI: region of interest
TSC: tuberous sclerosis complex
